# Identification of a Six-lncRNA Signature With Prognostic Value for Breast Cancer Patients

**DOI:** 10.3389/fgene.2020.00673

**Published:** 2020-07-24

**Authors:** Erjie Zhao, Yujia Lan, Fei Quan, Xiaojing Zhu, Suru A, Linyun Wan, Jinyuan Xu, Jing Hu

**Affiliations:** College of Bioinformatics Science and Technology, Harbin Medical University, Harbin, China

**Keywords:** long non-coding RNA, signature, prognosis, disease-free survival, breast cancer

## Abstract

Breast cancer (BRCA) is the most common cancer and a major cause of death in women. Long non-coding RNAs (lncRNAs) are emerging as key regulators and have been implicated in carcinogenesis and prognosis. In this study, we aimed to develop a lncRNA signature of BRCA patients to improve risk stratification. In the training cohort (GSE21653, *n* = 232), 17 lncRNAs were identified by univariate Cox proportional hazards regression, which were significantly associated with patients’ survival. The least absolute shrinkage and selection operator-penalized Cox proportional hazards regression analysis was used to identify a six-lncRNA signature. According to the median of the signature risk score, patients were divided into a high-risk group and a low-risk group with significant disease-free survival differences in the training cohort. A similar phenomenon was observed in validation cohorts (GSE42568, *n* = 101; GSE20711, *n* = 87). The six-lncRNA signature remained as independent prognostic factors after adjusting for clinical factors in these two cohorts. Furthermore, this signature significantly predicted the survival of grade III patients and estrogen receptor-positive patients. Furthermore, in another cohort (GSE19615, *n* = 115), the low-risk patients that were treated with tamoxifen therapy had longer disease-free survival than those who underwent no therapy. Overall, the six-lncRNA signature can be a potential prognostic tool used to predict disease-free survival of patients and to predict the benefits of tamoxifen treatment in BRCA, which will be helpful in guiding individualized treatments for BRCA patients.

## Introduction

Breast cancer (BRCA) is the second leading cause of cancer death among women. More than 268,000 new patients are diagnosed with BRCA each year and 41,760 patients will die from BRCA (DeSantis et al., 2019; Siegel et al., 2019). The current treatment for BRCA, which can improve survival of BRCA patients, includes mastectomy, hormone therapy (Early Breast Cancer Trialists Collaborative et al., 2011), surgery with adjuvant radiation therapy (Bradley and Mendenhall, 2018; Chargari et al., 2019), and chemotherapy (Oikonomou et al., 2019). Immunotherapy of BRCA patients is a recent emerging area of treatment (Greenlee et al., 2017; Jia et al., 2017; Adams et al., 2019). Although the TNM stage system is a valuable resource for the classification of BRCA patients, it does not predict the prognosis of patients. Therefore, the molecular markers need to be identified so that the survival of BRCA patients can be evaluated (Giuliano et al., 2017; Zhang et al., 2017).

Long non-coding RNAs (lncRNA, >200 nucleotides in length) are a class of non-coding RNAs transcribed from mammalian genomes (Yu et al., 2018). Some lncRNAs are found to be deregulated between cancer and normal tissues, such as BRCA (Liu et al., 2015), lung cancer (Jen et al., 2017), gastric cancer (Liu et al., 2017), and prostate cancer (Xu et al., 2018). Furthermore, lncRNAs have been confirmed to participate in diverse biological processes by acting as key regulators in cancers. Gupta et al. (2010) found that dysregulated *HOTAIR* increased cancer invasiveness and metastasis through dependence on PRC2, and lncRNA *HOXD-AS1* regulated the Rho GTPase activating protein 11A (ARHGAP11A), which resulted in induced metastasis (Lu et al., 2017). In recent years, some lncRNAs have been found to be biomarkers of predicting BRCA patient outcomes, such as lncRNA *BCYRN1* (Booy et al., 2017) and *HOTAIR* (Zhang et al., 2013a), which has attracted increasing attention.

In this study, we developed a six-lncRNA signature based on lncRNA expression, with the ability to predict disease-free survival of patients with BRCA, and we assessed its prognostic value in the training and validation cohorts. This signature had an independent prognostic value after adjusting for clinical factors. Furthermore, the lncRNA signature also significantly predicted survival of grade III and estrogen receptor (ER)-positive BRCA patients. Moreover, the signature predicted survival benefits of tamoxifen therapy in BRCA patients.

## Materials and Methods

### Study Samples

Breast cancer gene expression data generated by the Affymetrix HG-U133 Plus 2.0 microarray platform and corresponding clinical information were obtained from the publicly available GEO database^1^. To analyze the correlation of lncRNA expression with disease-free survival (DFS) for BRCA, we selected those data sets that included patients with survival status information. In total, 232 samples from GSE21653 (Sabatier et al., 2011a, b), 101 samples from GSE42568 (Clarke et al., 2013), and 87 samples from GSE20711 (Dedeurwaerder et al., 2011) were obtained. The GSE21653 data set was defined as the training cohort, and the GSE42568 and GSE20711 data sets were treated as the validation cohort. Another dataset, GSE19615 (*n* = 115) (Li et al., 2010), which contained 62 patients treated with tamoxifen, was obtained to validate the prognostic value of the signature for patients after hormone treatment. Detailed clinical information of patients with BRCA in this study is shown in Table 1.

**TABLE 1:** The Clinical and pathological characteristics of patients in four GEO cohorts.

Characteristic	GSE21653 (*n* = 232)	GSE42568 (*n* = 101)	GSE20711 (*n* = 87)	GSE19615 (*n* = 115)
Age (years)	55.0 (24.0–85.0)	56.9 (31.1–90.0)	53.8 (32.1–82.1)	53.0 (32.0–85.0)
Grade				
Grade I	39	10	13	23
Grade II	76	40	4	28
Grade III	117	51	70	64
ER status				
Positive	128	67	42	70
Negative	104	34	45	45
Median follow up (months)	51.8	66.0	71.4	64.0
Disease-free status				
Relapse	74	45	39	14
No relapse	158	56	48	101
Hormone therapy	−	−	−	
Tamoxifen	−	−	−	62
Arimidex	−	−	−	2
None	−	−	−	47
Unknown	−	−	−	4

### Microarray Data Processing and lncRNA Re-annotation

All the raw microarray data (CEL files) of BRCA patients were downloaded from the GEO database and background adjusted and normalized using the Robust Multichip Average (RMA) algorithm (Irizarry et al., 2003a, b) and “Affy” package (Gautier et al., 2004). The probe sequences of Affymetrix HG-U133 Plus 2.0 array were downloaded from the Affymetrix website^2^ and uniquely mapped to the human genome (hg19). Specific probes of lncRNAs were obtained by matching the chromosomal position of probes to the chromosomal position of lncRNA genes based on the annotations from GENCODE (release 23) according to the previous studies (Du et al., 2013; Zhou et al., 2015). When multiple probes were mapped to the same lncRNA, expression values of these probes were integrated using the median value to represent the expression value of the single lncRNA. As a result, 2,673 lncRNAs were obtained for further analysis.

### Identification of a Survival-Related lncRNA Signature Set Associated With Breast Cancer

A univariate Cox proportional hazards regression analysis was carried out to evaluate the association between expression levels of lncRNAs and patients’ disease-free survival in the training cohort. Only those lncRNAs with a *p*-value of <0.01 were considered statistically significant. We then conducted the least absolute shrinkage and selection operator (LASSO) penalized Cox proportional hazards regression analysis to select the prognostic markers of the above lncRNAs (Tibshirani, 1997; Zhang et al., 2013b). We created a risk-score formula by a linear combination of the expressions of these six lncRNAs, weighted by their respective Cox regression coefficients as follows (Zhang et al., 2012, 2013c):

$$RiskScore=\sum_{i=1}^{N}\left(Exp_{i}\times Coef_{i}\right)$$

where *N* is the number of prognostic genes, *Exp*_*i*_ is the expression value of the *i* gene, and *Coef*_*i*_ is the estimated regression coefficient of the *i* gene in the univariate Cox regression analysis. Using the median signature risk score in each cohort as the cutoff point, BRCA patients in every cohort were divided into low- and high-risk groups.

### Statistical Analysis

The association between the lncRNA gene expression and the patient’s survival was assessed by univariable Cox regression analysis. LASSO logistic regression analysis was used to identify the lncRNAs comprising the prognostic signature with non-zero coefficients in the training cohort using “glmnet” package (Friedman et al., 2010). Kaplan–Meier survival analysis and the log-rank test were used to compare the difference in disease-free survival between the high-risk group and low-risk group using the R package “survival.” Furthermore, we used Cox multivariate analysis to test whether the lncRNA signature was independent of patient age and histological grade. Hazard ratio (HR) and 95% confidence intervals (CI) were estimated by the Cox proportional hazards regression model. The time-dependent receiver operating characteristic (ROC) curves were used to compare the prognostic accuracy of the six-lncRNA signature for survival. Statistical significance was defined as two-tailed *p*-values being less than 0.05. All of the statistical analyses were performed using R program 3.5.2^3^ and Bioconductor.

## Results

### Identifying a Six-lncRNA Signature in the Training Cohort

**FIGURE 1:**
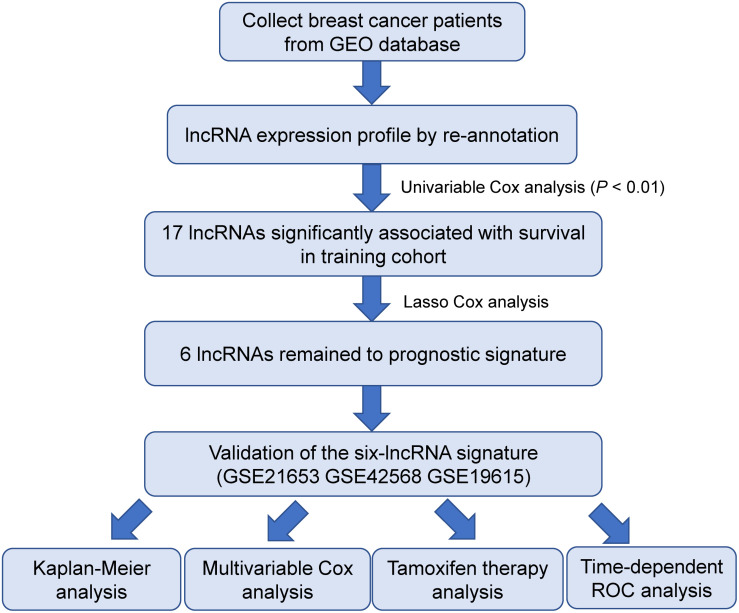
The workflow of identification and validation of the six-lncRNA signature.

As summarized in the workflow (Figure 1), we first performed an univariable Cox proportional hazards regression analysis to assess the association between lncRNA expression and disease-free survival of patients with BRCA in the training cohort. A set of 17 lncRNAs that were significantly correlated with patients’ survival (*p* ≤ 0.01, Table 2) was identified. We found six lncRNAs (*LINC00917*, *AL391840.1*, *TRIM52-AS1*, *AL355075.4*, *AC093802.2*, and *AC091544.4*) to comprise a prognostic signature using a LASSO-penalized Cox proportional hazards regression analysis for the above 17 lncRNAs with optimal tuning parameters. All six lncRNAs have positive coefficients, which indicates that their high expressions are associated with shorter survival. Finally, we calculated the signature risk score based on a linear combination of the expression levels of six prognostic lncRNAs, weighted by the coefficients derived from the univariable Cox regression analysis as follows: Risk Score = (1.6348 × expression value of *LINC00917*) + (1.7487 × expression value of *AL391840.1*) + (0.6661 × expression value of *TRIM52-AS1*) + (0.9439 × expression value of *AL355075.4*) + (1.1742 × expression value of *AC093802.2*) + (0.4818 × expression value of *AC091544.4*).

**TABLE 2:** The 17 lncRNAs that are significantly associated with the disease-free survival in the training cohort (*n* = 232).

Ensembl ID	lncRNA name	*P*-value	HR (95%CI of HR)
ENSG00000168367	LINC00917	0.0015	5.128(1.872−14.048)
ENSG00000215231	LINC01020	0.0046	10.021(2.036−49.326)
ENSG00000227467	LINC01537	0.0055	8.267(1.860−36.742)
ENSG00000224699	LAMTOR5-AS1	0.0091	1.762(1.151−2.698)
ENSG00000226754	AL606760.1	0.0034	2.184(1.294−3.685)
ENSG00000231533	AL391840.1	0.0054	5.747(1.678−19.686)
ENSG00000259001	AL355075.4	0.0082	2.570(1.276−5.176)
ENSG00000231312	MAP4K3-DT	0.0005	3.687(1.778−7.642)
ENSG00000231528	FAM225A	0.0027	2.220(1.318−3.738)
ENSG00000254887	AC010247.1	0.0054	3.369(1.432−7.923)
ENSG00000259889	AC093802.2	0.0008	3.236(1.619−6.468)
ENSG00000260337	AC091544.4	0.0036	1.619(1.170−2.240)
ENSG00000261292	AC110491.1	0.0036	2.041(1.262−3.301)
ENSG00000260027	HOXB7	0.0094	1.975(1.181−3.302)
ENSG00000248275	TRIM52-AS1	0.0041	1.947(1.235−3.068)
ENSG00000261357	AC099518.2	0.0015	7.756(2.189−27.479)
ENSG00000267317	AC027307.2	0.0022	2.219(1.331−3.699)

### The Six-lncRNA Signature Predicts Disease-Free Survival of Patients With Breast Cancer

**FIGURE 2:**
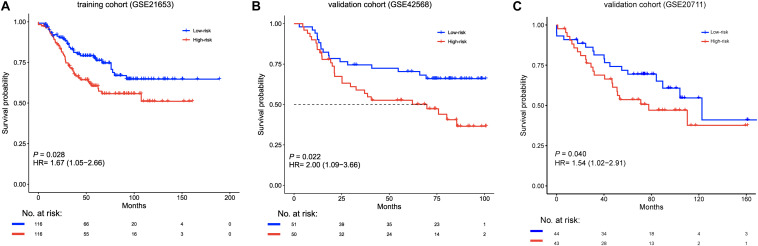
Kaplan–Meier survival curves of disease-free survival between high-risk (red) and low-risk (blue) patients in the **(A)** training cohort (GSE21653, *n* = 232), and the **(B,C)** validation cohort (GSE42568, *n* = 101; GSE20711, *n* = 87). The differences between the two curves were determined by the two-sided log-rank test. The number of patients at risk is listed below the survival curves. HR, hazard ratio.

We calculated the six-lncRNA signature risk score for each patient in the training cohort (GSE21653, *n* = 232). The patients were divided into a high-risk group (*n* = 116) and a low-risk group (*n* = 116) using the median risk score as the cutoff. Compared with the low-risk patients, the high-risk patients had shorter disease-free survival (median survival 62.4 months vs greater than 200 months, HR = 1.67, 95% CI = 1.05–2.66, *p* = 0.028, Figure 2A). The prognostic value of the six-lncRNA signature was then evaluated in the validation cohort (GSE42568, *n* = 101). The signature classified patients into two groups, including a high-risk group (*n* = 50) and a low-risk group (*n* = 51), based on the median risk score. The disease-free survival of the high-risk group was significantly shorter than that of the low-risk group (median survival 69.7 months vs greater than 100 months, HR = 2, 95% CI = 1.09–3.66, *p* = 0.022, Figure 2B). Similarly, in another validated cohort (GSE20711, *n* = 87), the high-risk group still had a poorer prognosis than the low-risk group (median survival 77.8 months vs 122.5 months, HR = 1.54, 95% CI = 1.02–2.91, *p* = 0.040, Figure 2C).

Next, we assessed whether the prognostic value of the six-lncRNA signature was independent of other clinical factors. We performed univariate and multivariate Cox proportional hazards regression analysis for factors, including age, ER status, histological grade, and the signature. In the training cohort, the high-risk six-lncRNA signature (HR = 1.789, 95% CI = 1.122–2.852, *p* = 0.015), grade III (HR = 3.174, 95% CI = 1.314–7.666, *p* = 0.010) and grade II (HR = 2.881, 95% CI = 1.181–7.028, *p* = 0.020) were significantly correlated with DFS of patients (Table 3). We found that the signature (HR = 2.327, 95% CI = 1.256–4.311, *p* = 0.007) and ER status (HR = 0.472, 95% CI = 0.234–0.877, *p* = 0.017) significantly independently predicted patients’ disease-free survival in the validation cohort GSE42568 (Table 3). Moreover, the six-lncRNA signature was also an independent prognostic factor associated with disease-free survival in the GSE20711 dataset (HR = 1.631, 95% CI = 1.037–3.105, *p* = 0.043). These results indicate that the six-lncRNA signature is an independent prognostic factor for BRCA patients’ disease-free survival.

**TABLE 3:** Multivariate analysis for the six-lncRNA signature of disease-free survival in cohorts.

Variables		Univariate analysis			Multivariate analysis		
		HR	95% CI	*P*-value	HR	95% CI	*P*-value
**Training set GSE21653 (*n* = 232)**							
Age		1.006	0.988−1.024	0.533	1.007	0.989-1.026	0.423
ER status							
	Positive vs Negative	0.670	0.424−1.059	0.087	0.772	0.468-1.272	0.310
Grade							
	Grade II vs Grade I	2.748	1.129−6.691	0.026*	2.881	1.181-7.028	0.020*
	Grade III vs Grade I	3.395	1.437−8.026	0.005*	3.174	1.314-7.666	0.010*
Six-lncRNA signature							
	High-risk vs Low-risk	1.674	1.052−2.664	0.030*	1.789	1.122-2.852	0.015*
**Validation set GSE42568 (*n* = 101)**							
Age		0.995	0.969−1.021	0.700	1.001	0.975-1.027	0.962
ER status							
	Positive vs Negative	0.439	0.243−0.793	0.006*	0.472	0.254-0.877	0.017*
Grade							
	Grade II vs Grade I	1.497	0.337−6.638	0.596	1.059	0.234-4.788	0.940
	Grade III vs Grade I	3.966	0.943−16.679	0.060	2.880	0.662-12.53	0.158
Six-lncRNA signature							
	High-risk vs Low-risk	1.998	1.092−3.655	0.025*	2.327	1.256-4.311	0.007*
**Validation set GSE20711 (*n* = 87)**							
Age		1.041	1.010−1.073	0.009*	1.043	1.013-1.075	0.005*
ER status							
	Positive vs Negative	0.554	0.286−1.070	0.079	0.637	0.308-1.316	0.223
Grade							
	Grade II vs Grade I	1.941	0.315−11.947	0.474	2.028	0.275-14.976	0.488
	Grade III vs Grade I	2.564	0.786−8.362	0.118	2.177	0.592-8.013	0.242
Six-lncRNA signature							
	High-risk vs Low-risk	1.539	1.021−2.905	0.040*	1.631	1.037-3.105	0.043*

### The Six-lncRNA Signature Predicts Survival of Patients During Diverse BRCA Groups

**FIGURE 3:**
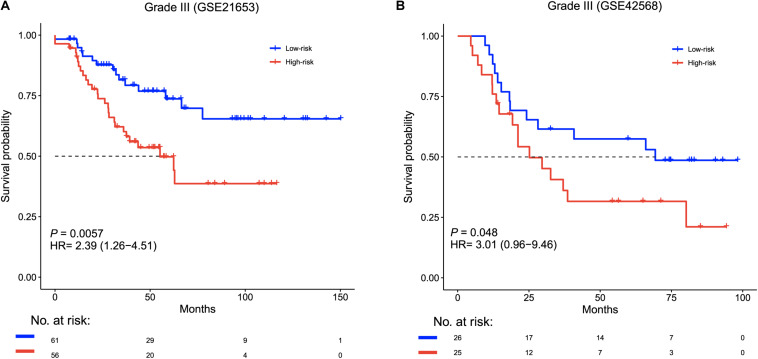
Survival analysis of grade III patients based on the six-lncRNA signature. Kaplan–Meier survival curves for grade III patients in **(A)** GSE21653 (*n* = 117) and **(B)** GSE42568 (*n* = 51).

We explored whether the six-lncRNA signature was effective for patients within different histological grades using a Kaplan–Meier survival analysis. For grade III patients, the signature significantly classified patients into two groups with distinctively different survival times (median survival 55.2 months vs greater than 150 months, HR = 2.39, 95% CI = 1.26–4.51, *p* = 0.0057, Figure 3A), including the high-risk group (*n* = 56) and the low-risk group (*n* = 61) in the training cohort. The signature showed a similar prognostic value for grade III patients in the validation cohort (median survival 25.2 months vs greater than 69.3 months, HR = 3.01 95% CI = 0.96–9.46, *p* = 0.048, Figure 3B). In grade I patients, there were no significant survival differences among the high-risk groups and the low-risk groups in two cohorts (Supplementary Figure S1A,B). A similar phenomenon was observed in grade II patients from the GSE21653 data set (Supplementary Figure S1C). However, in grade II patients from the GSE42568 data set, the high-risk and low-risk groups had significant survival differences (HR = 5.29, 95% CI = 1.17–23.9, *p* = 0.015, Supplementary Figure S1D).

**FIGURE 4:**
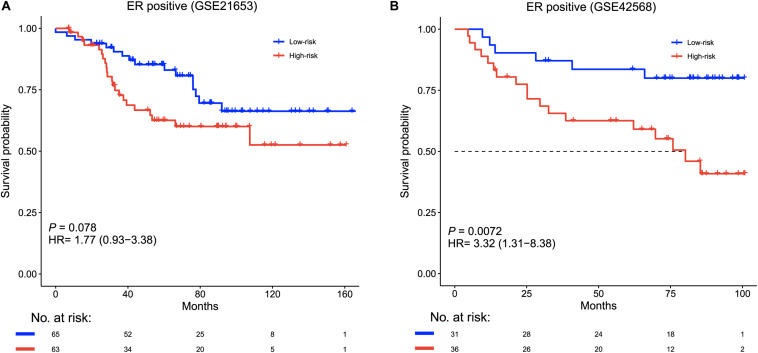
Survival analysis of ER-positive patients based on the six-lncRNA signature. Kaplan–Meier survival curves for ER-positive patients in **(A)** GSE21653 (*n* = 128) and **(B)** GSE42568 (*n* = 67).

Furthermore, Kaplan–Meier survival analysis was performed after patient stratification according to ER status. The ER-positive patients were divided into high-risk and low-risk groups. The high-risk ER-positive patients had shorter disease-free survival than low-risk ER-positive patients in the training cohort (HR = 1.77, 95% CI = 0.93–3.38, *p* = 0.078, Figure 4A) and the validation cohort (HR = 3.32, 95% CI = 1.31–8.38, *p* = 0.0072, Figure 4B). There were no significant survival differences between the high-risk and low-risk ER-negative patients in these two cohorts when using the same risk formula (Supplementary Figure S2).

### The Six-lncRNA Signature Predicts Patient Outcome After Tamoxifen Therapy

**FIGURE 5:**
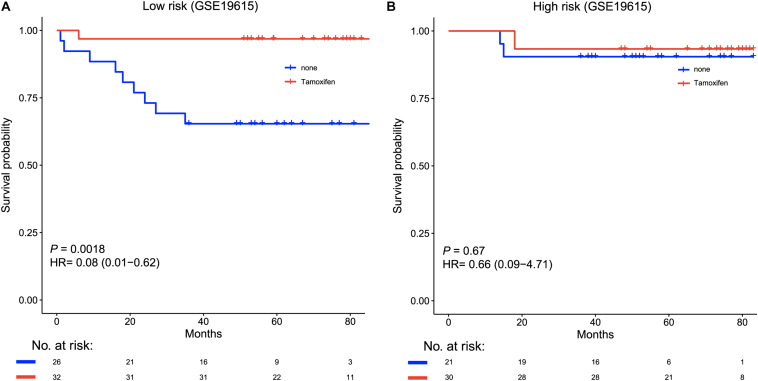
Kaplan–Meier curves of the disease-free survival according to low-risk or high-risk scores, stratified by tamoxifen therapy in an independent cohort (GSE19615, *n* = 115). **(A)** Patients with tamoxifen therapy (red) had significantly longer disease-free survival than patients without treatment (blue) in the low-risk group. **(B)** Patients who received tamoxifen therapy (red) and those who did not (blue) showed no survival differences in the high-risk group.

We further tested whether the six-lncRNA was useful to guide therapy in an independent cohort (GSE19615). In this cohort, there were 62 patients who received tamoxifen therapy and 47 who did not. We classified each patient into high- and low-risk groups based on the lncRNA signature risk score. Among the 58 low-risk patients, tamoxifen treatment could prolong the disease-free survival of these patients (HR = 0.08, 95% CI = 0.01–0.62, *p* = 0.0018, Figure 5A), while there were no significant survival differences between patients with and without tamoxifen therapy in the high-risk group (Figure 5B). This result revealed that tamoxifen treatment was only beneficial for low-risk BRCA patients.

### Comparison of the Survival Prediction Power Between Clinical Factors and the Six-lncRNA Signature

**FIGURE 6:**
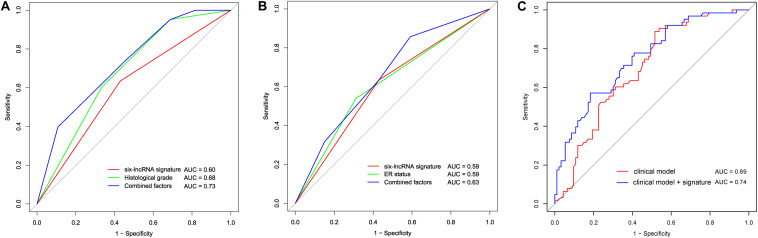
Comparison of sensitivity and specificity for survival prediction by the six-lncRNA signature, histological grade, and ER status. **(A)** The receiver operating characteristics (ROC) curves of the six-lncRNA signature, histological grade, and the combination of the two factors. **(B)** The ROC curves of the six-lncRNA signature, ER status, and the combination of the two factors. **(C)** The ROC curves of the clinical model and the clinical model combined with the six-lncRNA signature. AUC, the area under the curve.

To compare the sensitivity and specificity in survival prediction between clinical factors (histological grade and ER status) and the six-lncRNA signature, we performed a time-dependent ROC analysis in the training cohort. We also constructed a prognostic model by combining our signature with histological grade or ER status. There were no significant differences between histological grade and the lncRNA signature (*p* = 0.171). A similar result was found between the signature and ER status (*p* = 0.997). Moreover, for the histological grade, we observed that the histologic grade combined with the six-lncRNA signature (AUC = 0.73) had a higher area under the ROC curve than the histological grade alone (AUC = 0.68, Figure 6A). The six-lncRNA signature could also improve the prognostic accuracy of the ER status (0.63 vs 0.59, Figure 6B). In addition, for further clinical utility, we constructed a full clinical prognostic model by combining all clinical factors including age, histological grade, and ER status. After adding the six-lncRNA signature into the clinical prognostic model, the prediction accuracy of the model was effectively improved (0.74 vs 0.69, Figure 6C). These results suggest that our six-lncRNA signature can add a complementary value to known clinical factors.

## Discussion

In the current study, we developed and validated a prognostic six-lncRNA signature based on lncRNA expression, which stratified BRCA patients into two groups (high-risk group and low-risk group) with different disease-free survival. We demonstrated that this signature could predict the survival of grade III BRCA patients. The ER-positive patients who were classified as the low-risk group achieved better survival benefits. Furthermore, by using this signature, we can find a subgroup of patients who are likely to benefit from tamoxifen therapy. In sum, the six-lncRNA signature for BRCA patients may be a prognostic tool that is helpful in guiding individualized treatment of patients.

Histological classification of BRCA into grades I, II, and III, determines the treatment of BRCA patients (Cortes et al., 2012; Harris et al., 2016; Waks and Winer, 2019). The tumor cells of grade III cancer tend to grow more quickly and look different from normal breast cells (Wani et al., 2010). We observed that the six-lncRNA signature significantly predicted the survival of grade III BRCA patients. This finding suggests that this lncRNA signature predicted survival in patients with invasive cancer. In addition, we found that high-risk ER-positive BRCA patients had shorter disease-free survival than low-risk ER-negative patients. Some studies have confirmed that ER is an essential predictor for responding to therapy, such as tamoxifen therapy, in metastatic BRCA (Fisher et al., 1997).

Given the heterogeneity of cancer, reliable prognostic biomarkers are needed to identify patients who can benefit from therapy (Li et al., 2017; Zhang et al., 2018). There is growing research on several gene signatures to improve decision-making and individualization of BRCA therapy (Cronin et al., 2007; Cardoso et al., 2016; Yu et al., 2019). However, it is difficult to apply all of them for clinical management. Our prognostic signature could identify a group of patients at low risk, where the use of tamoxifen therapy led to significantly extended disease-free survival. This suggests that our signature may hold special clinical value by separating responders to tamoxifen treatment, from non-responders, independent of pathological stage. Such separation could spare non-responders from therapy that is not beneficial and could promote the exploration of more effective therapeutic regimens.

The six-lncRNA (*LINC00917*, *AL391840.1, TRIM52-AS1*, *AL355075.4*, *AC093802.2*, and *AC091544.4*) signature in BRCA suggests that lncRNAs can be used as prognostic factors for the survival of patients. To avoid the influence of protein-coding genes, we annotated these probes with protein-coding genes, and found that only one lncRNA overlapped with protein-coding gene RPPH1. This gene had no predictive performance for survival, whether by itself or in combination with other lncRNAs (*p* = 0.39 and 0.16 respectively, Supplementary Figure S3). In addition, among these lncRNAs, *TRIM52-AS1* was dominantly up-regulated in triple-negative breast cancer (TNBC) tissues compared to non-TNBC tissues by a RT-PCR (Lv et al., 2016). Moreover, another study found that the overexpression of TRIM52-AS1 suppressed cell migration and proliferation and induced cell apoptosis in renal cell carcinoma (Liu et al., 2016). However, these six lncRNAs have not been studied in BRCA. Thus, this is a novel study on the association between lncRNA expression and the disease-free survival of patients with BRCA.

Although the signature demonstrated an accurate survival prediction, several limitations should be noted. Because the sample size of our study was limited, large-scale cohort studies should be performed to investigate the prognostic value of this six-lncRNA signature. In addition, we only used a bioinformatics method to predict the six-lncRNA signature in BRCA, thus, further *in vitro* or *in vivo* experiments need to be conducted. Third, we investigated the efficacy of tamoxifen therapy in a low-risk BRCA group, thus more examinations are required to evaluate its efficacy and safety.

In conclusion, the six-lncRNA signature that we identified predicted the disease-free survival of patients with BRCA. This signature also predicted the survival of grade III and ER-positive patients. Furthermore, our findings revealed that the six-lncRNA signature could predict the benefits to patients treated with tamoxifen therapy. Further validation studies are needed to test the prognostic power of this signature before it is used clinically.

## Data Availability Statement

All datasets used in this study were publicly available from the GEO database (https://www.ncbi.nlm.nih.gov/geo) at accession numbers GSE21653, GSE42568, GSE20711, and GSE19615.

## Author Contributions

JH conceived the idea and conceptualized the study. EZ, YL, FQ, and JX conducted the bioinformatics analysis and interpreted the results. EZ and JX collected and pre-processed data. EZ, YL, SA, and LW generated the figures and tables. EZ and YL wrote the manuscript. JH and JX supervised the whole study process and revised the manuscript. All authors have read and approved the final version of manuscript.

## Conflict of Interest

The authors declare that the research was conducted in the absence of any commercial or financial relationships that could be construed as a potential conflict of interest.

## Footnotes

1 https://www.ncbi.nlm.nih.gov/geo/

2 http://www.affymetrix.com/

3 https://www.r-project.org/
