# Supplementary material for: Identification of a Six-lncRNA Signature With Prognostic Value for Breast Cancer Patients
Source: Front Genet. 2020 Jul 24;11:673. doi: 10.3389/fgene.2020.00673 (PMC7396575; doi:10.3389/fgene.2020.00673)
Supplement: Supplementary file 1 [file Data_Sheet_1.docx]

Supplementary Material

**Supplementary Figures**


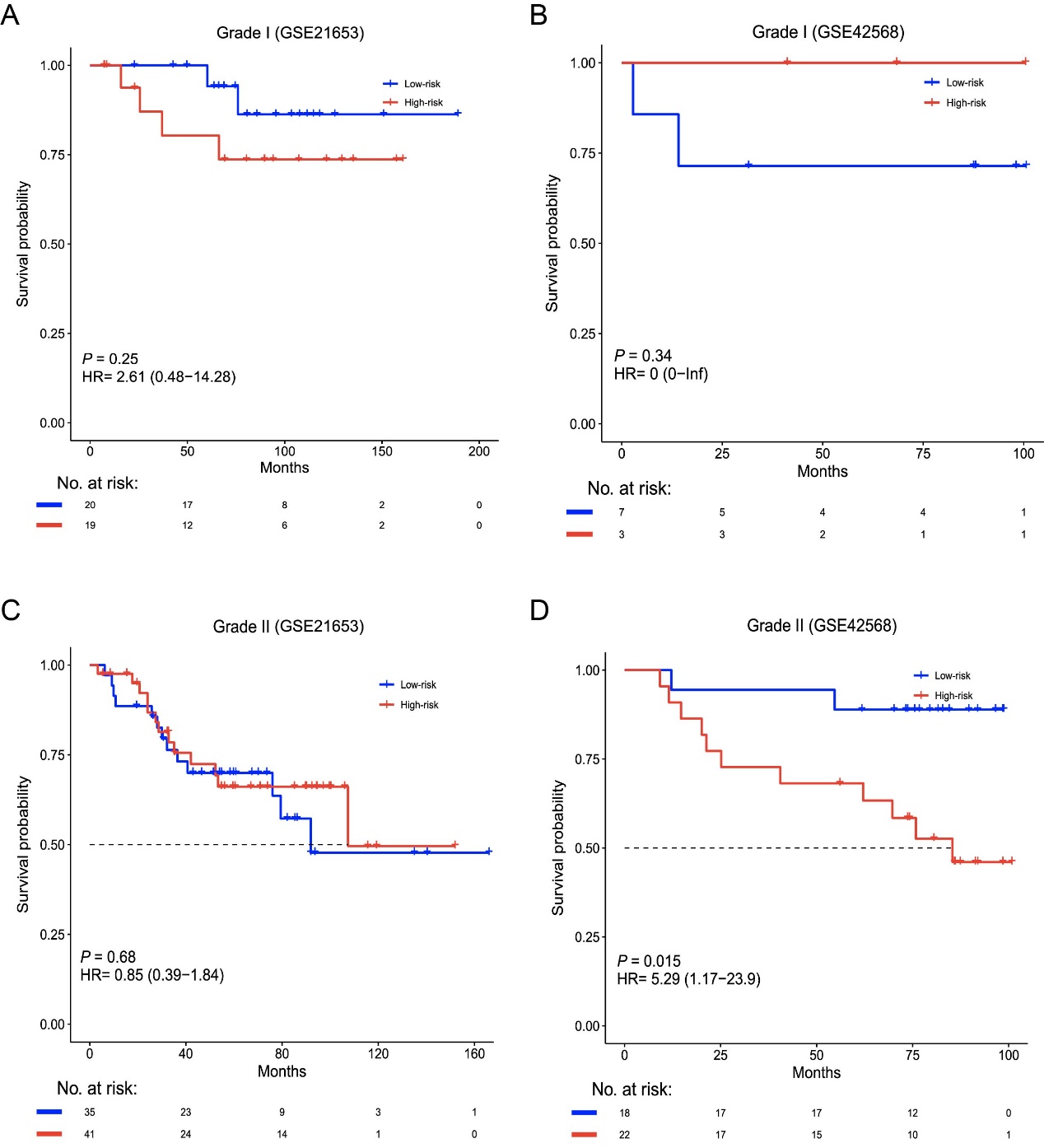


**Figure S1.** Survival analysis of grade I and II patients based on the six-lncRNA signature. Kaplan-Meier survival curves for grade I patients in (A) GSE21653 (n = 39) and (B) GSE42568 (n = 10). Kaplan-Meier survival curves for grade II patients in (C) GSE21653 (n = 76) and (D) GSE42568 (n = 40).


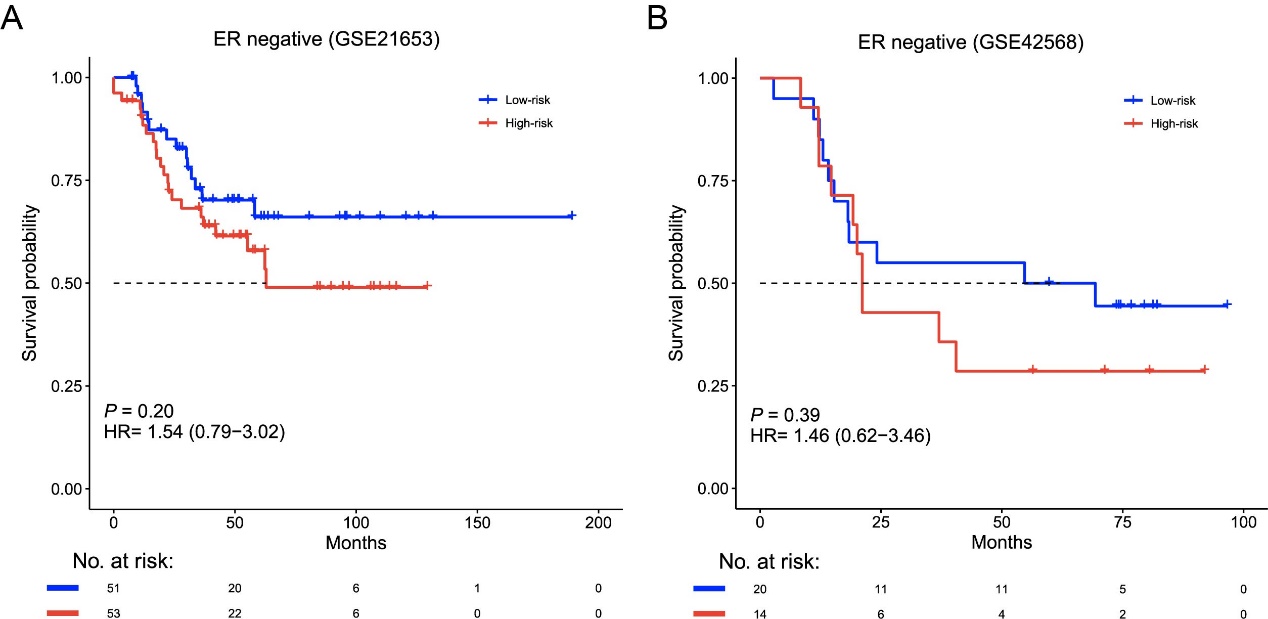


**Figure S2.** Survival analysis of ER negative patients based on the six-lncRNA signature. Kaplan-Meier survival curves for ER negative patients in (A) GSE21653 (n = 104), (B) GSE42568 (n = 34).


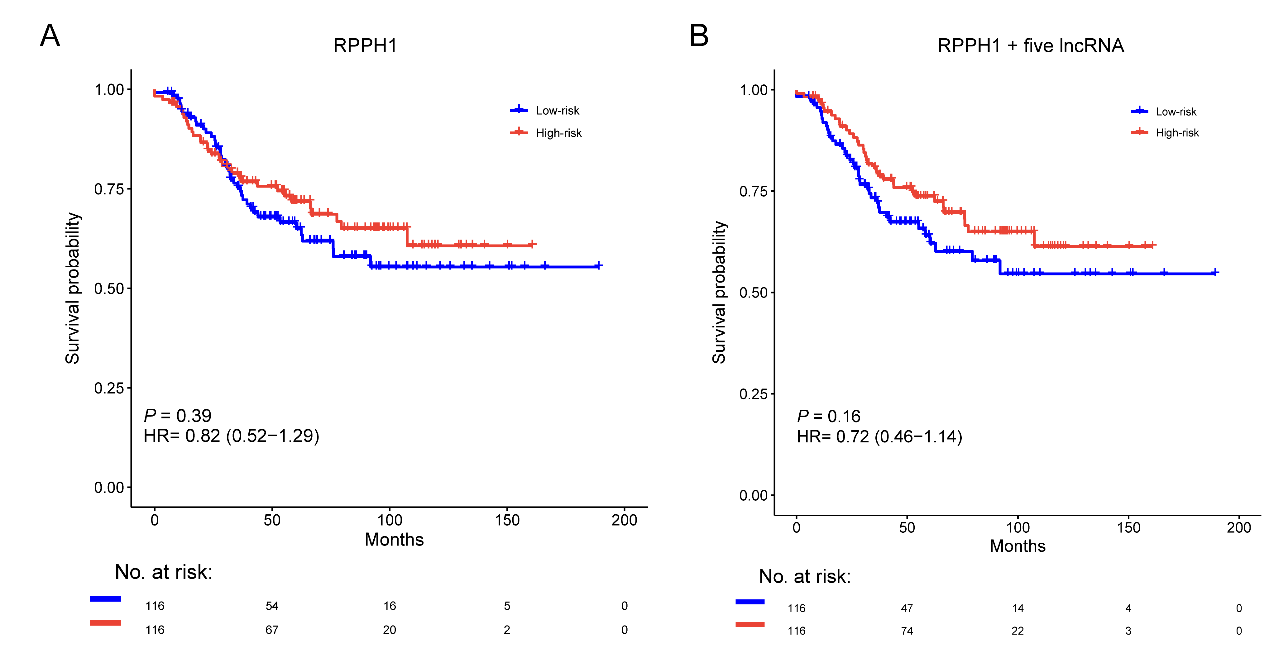


**Figure S3.** Survival analysis of breast cancer patients based on the *RPPH1* expression (A) and combining *RPPH1* with the left five lncRNAs (B) in GSE21653 (n = 232).
